# Release of Nitrogen and Phosphorus from Poultry Litter Amended with Acidified Biochar

**DOI:** 10.3390/ijerph8051491

**Published:** 2011-05-11

**Authors:** Sarah A. Doydora, Miguel L. Cabrera, Keshav C. Das, Julia W. Gaskin, Leticia S. Sonon, William P. Miller

**Affiliations:** 1 Department of Crop and Soil Sciences, The University of Georgia, 3111 Miller Plant Sciences Building, Athens, GA 30602, USA; E-Mails: sdoydora@uga.edu (S.A.D.); wmiller@uga.edu (W.P.M.); 2 Department of Biological and Agricultural Engineering, Driftmier Engineering Center, The University of Georgia, Athens, GA 30602, USA; E-Mails: kdas@engr.uga.edu (K.C.D.); jgaskin@engr.uga.edu (J.W.G.); 3 Soil, Plant, and Water Laboratory, 2400 College Station Road, Athens, GA 30602, USA; E-Mail: lsonon@uga.edu

**Keywords:** acidified biochar, poultry litter, inorganic nitrogen, inorganic phosphorus, ammonia volatilization

## Abstract

Application of poultry litter (PL) to soil may lead to nitrogen (N) losses through ammonia (NH_3_) volatilization and to potential contamination of surface runoff with PL-derived phosphorus (P). Amending litter with acidified biochar may minimize these problems by decreasing litter pH and by retaining litter-derived P, respectively. This study evaluated the effect of acidified biochars from pine chips (PC) and peanut hulls (PH) on NH_3_ losses and inorganic N and P released from surface-applied or incorporated PL. Poultry litter with or without acidified biochars was surface-applied or incorporated into the soil and incubated for 21 d. Volatilized NH_3_ was determined by trapping it in acid. Inorganic N and P were determined by leaching the soil with 0.01 M of CaCl_2_ during the study and by extracting it with 1 M KCl after incubation. Acidified biochars reduced NH_3_ losses by 58 to 63% with surface-applied PL, and by 56 to 60% with incorporated PL. Except for PH biochar, which caused a small increase in leached NH_4_ ^+^-N with incorporated PL, acidified biochars had no effect on leached or KCl-extractable inorganic N and P from surface-applied or incorporated PL. These results suggest that acidified biochars may decrease NH_3_ losses from PL but may not reduce the potential for P loss in surface runoff from soils receiving PL.

## 1. Introduction

Georgia is the top broiler-producing state in the United States of America, with a production of 1.4 billion birds in 2008 [1]. Assuming an average litter production of 1.5 kg per bird [2,3], Georgia is estimated to have produced 2.1 million Mg of poultry litter (PL) in 2008. Consisting primarily of poultry manure, bedding material, feathers, and some wasted feed, PL provides both macro and micronutrients to crops. Much of this PL is surface applied to pastures and no-till fields, or incorporated into the soil in conventional-till fields as a fertilizer. While this material offers an inexpensive fertilizer source particularly for nitrogen (N) and phosphorus (P), its N value decreases when N is lost as ammonia (NH_3_) gas, especially from surface applications. This process, known as NH_3_ volatilization, accounts for 4 to 60% of the total N lost from PL under laboratory conditions [4–6]. Ammonia loss from PL is important not only agronomically, but also environmentally. Deposition of NH_3_ from the atmosphere can lead to N loading of lakes, indirect acidification of soils of low buffering capacity through nitrification, and damage of sensitive crops such as tomato, cucumber, and conifers [7–10]. In addition to the potential NH_3_ impact, applying PL to soil may contaminate surface runoff with P [11]. Amending PL with acidified biochar may be useful to reduce such losses of N and P from PL. Reduction in the amounts of volatilized NH_3_ may be achieved with biochar by decreasing the pH of the litter and by providing more exchange sites for ammonium (NH_4_ ^+^). Reduction in the amounts of lost P may be achieved by the biochar’s capacity to adsorb phosphate [12].

Biochar is a black solid byproduct produced when biomass residues are converted to liquid and gaseous fuel through pyrolysis. It is composed primarily of polyaromatic carbon (C) [13–16], which suggests high resistance against decomposition [17–20]. Biochar has gained increasing interest as one of the means for sequestering carbon dioxide (CO_2_) from the atmosphere. Lehmann [21] argues that when combined with bioenergy production, heating plant biomass can be a clean technology while at the same time sequestering carbon in biochar. When applied to soils, this byproduct has an estimated residence time of hundreds to thousands of years [19,21,22]. Adding to its refractory nature towards microbial breakdown, biochars are also reported to increase the cation exchange capacity (CEC) of soils [23,24]. The presence of biochars has been associated with the enhanced nutrient retention of Terra Preta soils that were formed originally from nutrient-poor, leaching-prone soils in the Central Amazonia [20,23]. The retentive property of biochars is also one of the major factors ascribed to better crop production with inorganic or organic fertilizer plus biochar combinations over inorganic or organic fertilization alone under tropical conditions [12,20,25,26].

Considering the environmental and economic benefits of renewable bioenergy over conventional burning of fossil fuels [21,27–30], it is anticipated that thermochemical generation of bio-fuel will be one of the alternatives to petroleum. In view of the anticipated increase in the supply of biochars in the future, utilizing this byproduct as a means for improving the retention capacity of poultry litter for nutrients such as N and P warrants an investigation.

The effect of biochar on the release of inorganic N and P has not been well investigated particularly in PL-fertilized soils. Assessment of the release of potentially available N and P is important for the resulting impact of acidified biochar-amended PL on both plants and the environment. Therefore, the objectives of this study were to evaluate the effect of acidified biochars on NH_3_ volatilization and release of inorganic N and P from surface-applied or incorporated PL.

## 2. Experimental Section

### 2.1. Biochar Production and Acidification

Biochars were produced from pine chips (PC) and pelletized peanut hull (PH) residues by slow pyrolysis under N_2_ gas at a peak temperature of 400 °C and 1-h residence time. These pyrolysis conditions generated two types of biochar: PC and PH. The biochars were ground and sieved through a 53-μm mesh. Two 25-g subsamples of each biochar were treated with acid. Each 25-g subsample was shaken with 250 mL of 0.5 N HCl for 30 min, the suspension was allowed to stand for 24 h, and then filtered through a 0.45-μm membrane (GE Water and Process Technologies, Trevose, PA). The biochar collected on the filter was oven-dried at 65 °C for 48 h, and the two subsamples of each biochar were thoroughly mixed together.

### 2.2. Soil and Poultry Litter Sampling and Characterization

Soil samples were collected from the upper 10 cm of a pasture that had received many years of PL application at the Central Research and Education Center of the University of Georgia (33° 24′ N, 83° 29′ W, elevation 150 m). The dominant soil series in that pasture was Cecil (fine, kaolinitic, thermic Typic Kanhapludults). The soil samples were air-dried, passed through a 2-mm sieve, and mixed with acid-washed sand at a 1:1 (w/w) ratio. The acid-washed sand was added in order to facilitate leaching of inorganic N and P at determined times during the incubation study. A PL was collected from a PL stack remaining after application of PL to the sampled pasture in Spring 2008.

### 2.3. Incubation Experiment

A wet mixture of acid-washed sand and soil (64.5 g dry-mixture equivalent, 0.20 g H_2_O g^−1^ dry mixture), hereinafter referred to as soil to avoid confusion, was packed to a depth of 1 cm in plastic funnels (7.4 cm ID) lined with a 0.45-μm filter membrane at the bottom. The 1-cm depth of packing was selected to achieve a bulk density of 1.5 g cm^–3^, which is typical of soils in the sampled pasture [31]. Twelve funnels were prepared for each study to accommodate three replications of four treatments in a completely randomized design. The four treatments were: T0 (no PL or biochar), T1 (2.09 g dry-weight equivalent PL, 0.33 g H_2_O g^−1^ dry PL), T2 (2.09 g dry-weight equivalent PL + 2.09 g dry weight PC), and T3 (2.09 g dry-weight equivalent PL + 2.09 g dry weight PH). The application rate of PL was based on 200 kg PL-N ha^−1^ (or 4,781 kg PL ha^−1^) on an area basis while the application rate of the biochars was based on a 1:1 ratio PL:biochar (or 4,781 kg biochar ha^−1^). These treatments were tested in two separate studies, one with surface applied PL and one with incorporated PL. Treatments with acidified biochars and PL were mixed thoroughly before being applied to soil. For the surface-applied incubation, the treatments were directly applied on the surface of the packed soil, whereas for the incorporated incubation, the treatments were thoroughly mixed with the soil before packing the whole mixture in the funnel. Other than the manner of application of the treatments, the two incubation studies were conducted using the same procedures throughout the incubation period.

Each funnel (experimental unit) was placed in a flow-through system set up inside an incubator at 20 °C. The system circulated humidified air (90% relative humidity) at a rate of 0.86 L min^−1^ for 21 d. Ammonia lost was trapped by bubbling the air leaving each funnel through 50 mL of 0.1 N H_2_SO_4_. The acid traps were replaced at 1, 3, 5, 7, 10, 14 and 21 d of incubation to avoid saturation of the traps with NH_3_. On the 14th day, each funnel was taken out of the incubator and leached with 150 mL of 0.01 M CaCl_2_, in 30-mL increments every 1.5 h. We used 0.01 M CaCl_2_ instead of deionized water to maintain the flocculated state of soil particles and prevent soil dispersion. After the last addition of 0.01 M CaCl_2_, 50 mL of N- and P-free solution (66.81 mg Ca L^–1^, 39.22 mg Mg L^–1^, 21.72 mg S L^–1^, and 119.70 mg K L^–1^) was added in 10-mL increments every 30 min and collected in the same container used for the 0.01 M CaCl_2_ leachate. Leaching the samples with the above nutrient solution was done to replenish the nutrients that may have been removed by the CaCl_2_ and to remove the entrained Cl^–^ remaining from the CaCl_2_ solution. After leaching, the funnels were incubated again at the same temperature and airflow rate. On the 21st day, the funnels were leached again with the same solutions in the same manner. After this second leaching, the funnel contents were extracted with 1 M KCl at a 1:10 soil-solution ratio [32].

### 2.4. Analysis of Samples

The NH_3_ traps, leachate samples, and KCl extracts were analyzed for NH_4_ ^+^-N colorimetrically [32] at 667 nm using a UV spectrophotometer (Shimadzu Corp., Kyoto, Japan). In leachate and KCl extracts, NO_3_ ^–^-N and PO_4_ ^–3^-P analyses were carried out with an autoanalyzer (Alpkem Corp., College Station, TX) following the ascorbic acid method for P [33] and the nitrate-reduction method [32] for NO_3_ ^–^-N. The soil and acidified biochar samples were analyzed for pH in water (1:30 sample: water ratio), initial inorganic N [32] and P at 1:10 biochar-1 M KCl ratio [33], total C and total N by dry combustion [34], total P using Kjehldahl digestion [35], CEC in 1 M NH_4_OAc at pH 7 [36], and H^+^ buffering capacity. The H^+^ buffering capacity determination for the acidified biochars and the soil was done by suspending a known amount of these materials in deionized water, and titrating it with a known concentration of NaOH up to pH 7.5 under automatic stirring. After titrating, the suspension was allowed to shake for 24 h, measured for equilibrium pH and the titration was repeated again. The cycle was repeated 4 more times (for a total of 5 days). The equilibrium pH used was the one obtained after each 24-h shaking. The H^+^ buffering capacity was estimated by calculating the amount of base used per unit weight of the materials used per unit pH change between the titrated samples and the untitrated (control) samples. The cumulative H^+^ buffering capacity added over the 5-day period (Table 1) is important because each H^+^ released to the soil solution gives rise to a negative charge that increases CEC. An increase in the CEC may lead to more adsorption of NH_4_ ^+^ thereby decreasing the ammoniacal N susceptible to volatilization. The PL samples were also analyzed for the same parameters as soil and acidified biochars except for CEC and buffering capacity, which were not measured.

### 2.5. Calculations and Statistical Analysis

Inorganic N and P in the leachate (amounts from the two leaching events combined) and in the final KCl extracts were reported as the amounts removed from each experimental unit (funnel) per gram of soil after correcting for the amounts measured in the control treatment (T0). The amounts of NH_3_ volatilized were estimated by subtracting the amounts of NH_3_ volatilized from soil alone (T0) from the rest of the treatments and expressing the results as μg N g^−1^ soil. Total inorganic N and P released from the treatments were calculated by adding the amounts from the leachates, from the KCl extracts, and from the NH_3_ traps for inorganic N, and those amounts from the leachates and KCl extracts for inorganic P. Total inorganic N and P released were also reported as amounts per gram of soil. All of the parameters evaluated in this study were statistically analyzed as one-way structure analysis of variance (ANOVA) using PROC GLM in SAS version 9.1 [37]. All treatment mean comparisons were done based on LSMEAN differences at 0.05 level of significance.

## 3. Results and Discussion

Surface-applied PL volatilized 226 μg N g^−1^ soil whereas PLs amended with acidified biochars lost only 83 to 95 μg N g^−1^ after 21 d (Table 2). Thus, adding acidified biochars reduced NH_3_-N loss by 58 to 63%. No differences were observed between PLs amended with PC and PH. The reduction in NH_3_ loss caused by the addition of biochar may have been due to a reduction in the pH of the PL + biochar mixture (Table 3), an increase in the hydrogen buffering capacity, and an increase in the cation exchange capacity (Table 1).

Poultry litter contains uric acid, which is converted to urea by the enzyme uricase. Urea is in turn hydrolyzed to ammoniacal N by the enzyme urease, with the process consuming H^+^ ions and raising pH [38]. Volatilization of NH_3_ is a pH-dependent process, with conversion of aqueous NH_4_ ^+^ into NH_3_ starting around pH 7 [39]. Thus, decreasing the pH of the original PL from 8.55 to values of 7.26 to 7.39 by the addition of biochars (Table 3) likely decreased the conversion of ammoniacal N to NH_3_, which helped reduce losses. In addition, the biochars provided H^+^ buffering capacity that likely helped resist increases in pH caused by urea hydrolysis (Table 1). Furthermore, biochars may have kept the amounts of volatilized NH_3_ at lower levels by also retaining some of the NH_4_ ^+^ on their exchange sites. If all of the exchange sites on the biochars were totally occupied by NH_4_ ^+^, the theoretical maximum reduction in NH_3_ volatilized from surface-applied PL could have been 79 and 71 μg N g^−1^ soil by PC and PH, respectively (for PC: 2.09 g PC × 1 kg PC 1000 g^−1^ PC × 17.4 cmol + kg^−1^ × 0.14 g N cmol^−1^ × 1000 mg N g^−1^ N × 1 experimental unit (64.5 g soil) ^−1^ × 1000 g soil kg^−1^ soil = 79 mg N kg^−1^ = 79 μg N g^−1^ soil). However, these values could only account for about 55% (for PC: 79/(226 – 83) × 100 = 55%; for PH: 71/(226 – 95) × 100 = 54%) of the actual reduction in NH_3_ loss observed by the addition of biochar in the surface-applied study (Table 2). Therefore, it is likely that the addition of biochar reduced NH_3_ losses by a combination of pH decrease and increases in H^+^ buffering and cation exchange capacities of the PL + biochar mixture.

Incorporated PL without biochar lost 97 μg N g^−1^ soil through NH_3_ volatilization (Table 2). Although the treatments of surface-applied and incorporated PL were not applied in the same study and therefore cannot be statistically compared, it can be clearly seen that incorporated PL led to a smaller amount of NH_3_ loss than surface-applied PL (226 μg N g^−1^ soil). The smaller loss with incorporated treatments was likely due to (1) a lower amount of total released N (Table 2), possibly due to immobilization, and (2) a larger decrease in pH (Table 3), which would have reduced the amount of NH_3_ in solution. For incorporated PL, taking into account the pH difference observed between the soil-alone treatment (pH = 6.69 from Table 1) and the pH of soil + PL (pH = 6.94 from Table 3), it is possible to use the H^+^ buffering capacity (Table 1) of the soil to calculate the amount of poultry litter N that would have been retained by the soil as NH_4_ ^+^-N. Based on a H^+^ buffering capacity of 4 cmol H^+^ kg^−^**^1^** pH^−^**^1^** (Table 1) an increase in pH of 0.25 units would have created 1 cmol of negative charge kg^−^**^1^** (0.25 pH units × 4 cmol negative charge kg^−^**^1^** pH^–1^), which could have retained a maximum of 140 μg N g^−1^ soil (1 cmol NH_4_ ^+^-N kg^−1^ × 0.14 g N cmol^−1^ × 1000 mg g^−1^ = 140 mg N kg^−1^ = 140 μg N g^−1^). However, considering the possibility that (1) some of the released acid from the soil may have been consumed by the alkaline buffering capacity of PL (which was not determined in this study), and (2) some of the created CEC may have been occupied by other cations in the PL, the soil may have retained less than 140 μg N g^−1^ soil. The difference between what could be potentially volatilized (based on the losses observed in the unamended surface-applied PLs [226 μg N g^−1^ soil]) and the estimate of the amount of N that the soil could retain (<140 μg N g^−1^ soil) would indicate that >86 μg N g^−1^ soil (226 – 140 = 86) could still be lost from the incorporated unamended PL. This estimate is not far from the 97 μg N g^−1^ soil that was actually volatilized from unamended incorporated PL. Therefore, insufficient H^+^ buffering capacity from the soil used in this study may have been the reason why some NH_3_ was still volatilized from unamended PL even under incorporated incubation.

When acidified biochars were added, incorporated PL lost 39 to 43 μg N g^−1^ as NH_3_ after 21 d (Table 2), which corresponds to reductions of 56 to 60% when compared to unamended PL. The lack of differences in NH_3_ loss among biochar-amended PLs may have been due to the biochars providing similar H^+^ buffering capacities (Table 1).

Total amounts of leached inorganic N tended to be greater from surface-applied and incorporated PL with acidified biochars than without these amendments, particularly for PH (Table 2). This was probably due to the reduction in NH_3_ loss caused by the biochar addition, which would have left more inorganic N in the soil susceptible to leaching. When analyzing the constituents of inorganic N leached (NH_4_ ^+^-N or NO_3_ ^−^-N), however, large variability in the data prevented clear detection of an increased amount of leached NH_4_ ^+^ (Table 4). Leached amounts of NH_4_ ^+^-N or NO_3_ ^−^-N in all PL + biochar treatments are assumed to be derived from PL because the initial amounts of inorganic N present in the acidified biochar were very low compared to that of PL (Table 1). It should be noted that the large amounts of leached NH_4_ ^+^ may have been caused by the use of 0.01 M CaCl_2_ as leaching solution because Ca_2_^+^ (even at low concentrations) could have displaced cations from exchange sites. The use of deionized water as leaching solution would have likely resulted in lower amounts of leached NH_4_ ^+^, but soil dispersion caused by deionized water prevented us from using it.

Inorganic P leached from biochar-amended PL was not significantly different from that of the unamended PL in surface or incorporated incubations (Table 2). In general, incorporated litter with or without acidified biochars tended to have more inorganic P leached compared to surface-applied treatments, although these differences could not be tested statistically because the surface-applied and incorporated studies were conducted separately. As shown in Table 3, litters unamended or amended with acidified biochars in the presence of soil (in the same proportion as those in the incorporated incubation) had lower starting pHs compared to those that were measured without soil (as in surface incubation). Lower pHs of incorporated compared to surface-applied litters may have caused more of the inorganic P from PL to solubilize [40] and become available for leaching.

Acidified biochars had no significant effect on KCl-extractable NH_4_ ^+^-N, NO_3_ ^–^-N (data not shown) and PO_4_ ^–3^-P (Table 2) in surface-applied and incorporated PLs. Release of total inorganic N from biochar-amended PLs also showed no significant difference from unamended PL in surface-applied or incorporated incubations (Table 2). For surface-applied PLs, 62 to 68% of the total amounts of the released inorganic N (totaled from volatilized, leached and extracted N) was apparently derived from the initial inorganic N of the PL, whereas for incorporated PLs all the released inorganic N apparently came from its initial inorganic N. Negative values for the percentage of organic N mineralized from incorporated PL (data not shown) indicates immobilization of its mineralized N or possibly even some of its initial N. This may be the reason for lower amounts of total inorganic N released from incorporated compared to surface-applied PL. Nitrogen immobilization may have been due to the fact that the soil used in the study was collected from a pasture and although it was passed through a 2-mm sieve, it still contained very small particles of plant residues.

Several workers have reported that crop performance is enhanced when inorganic or organic fertilizers are combined with charcoal. This has been assumed to be partly due to the enhanced nutrient retention capacity of the soil with charcoal [12,20,26,41] particularly for leaching-prone soils in the tropics. For example, in their lysimeter experiment with rice, Lehmann *et al.* [42] observed increased proportion of N taken up by the plants relative to what was lost to leaching when charcoal was added to inorganic or organic fertilizers. In our study, however, acidified biochars did not reduce the amounts of leached or extracted inorganic N. This may have been due to the use of 0.01 M CaCl_2_ as leaching solution or to the relative low concentration of biochar used. The amount of biochar used in our study was only 3.2% of the soil weight in each experimental unit (2.09 g biochar/64.5 g soil × 100 = 3.2%) compared to 20% of the soil weight used by Lehmann *et al.* [42].

Increased P availability has been reported in the presence of biochar [43–46]. The mechanisms suggested for biochar influence on P availability are change in soil pH, which then influences the interaction of P with other cations, or enhanced retention through anion exchange [46]. In this study, however, acidified biochar at the applied rates did not have a significant effect on soil P retention

## 4. Conclusions

Acidified biochars reduced NH_3_ losses by 58 to 63% in surface-applied PL and by 56 to 60% in incorporated PL. Adding acidified biochars to PL generally had no effect on the amounts of leached NH_4_ ^+^-N, NO_3_ ^–^-N and KCl-extractable N from surface-applied and incorporated PLs, except for a small increase in leached NH_4_ ^+^ from PL + PH in the incorporated incubation. If leached and extractable N is considered potentially available to plants, then the lack of a biochar effect may be considered a positive effect for soils that are not susceptible to leaching. However, acidified biochars did not affect the amounts of leached- or KCl-extracted P, which indicates that these biochars may not be useful to reduce P loading in runoff from pastures receiving surface-applied PL.

## Figures and Tables

**Table 1.** Selected chemical properties of soil, poultry litter, and acidified biochars.

**Sample**	**pH**	**NH_4_^+^ (μg N g ^−1^)**	**NO_3_^−^ (μg N g^−1^)**	**PO_4_^−3^ (μg P g ^−1^)**	**Buffering capacity cmol H^+^ kg ^−1^ pH ^−1^**	**Total C, g kg ^−1^**	**Total P, μg g ^−1^**	**Total N, μg g ^−1^**	**CEC, cmol + kg ^−1^**
Soil†	6.69 b‡	10 b	51 b	11 b	4 b	22 c	724 b	2,027 b	6.4 c
PL	8.62 a	9,378 a	633 a	340 a	ND	353 b	16,238 a	41,833 a	ND
PC	2.54 c	1 b	0 b	11 b	28 a	608 a	18 b	1,372 b	17.4 a
PH	2.55 c	1 b	0 b	36 b	35 a	625 a	126 b	1,835 b	15.7 b

Legend for Table 1: PL refers to poultry litter; PC and PH refer to biochar from pine chips and peanut hulls, respectively. ND means not determined.

‡ Within each column, different letters (a,b,c) mean significant difference between treatments at 0.05 level of significance.

† means soil + sand together.

**Table 2.** Total Inorganic N and P released from surface-applied or incorporated poultry litter with or without acidified biochars (control treatment subtracted).

**Treatments**	**Inorganic N, μg g ^−1^**				**Inorganic P, μg g ^−1^**		

	**Volatilized**	**Leached**	**Extracted**	**Total**	**Leached**	**Extracted**	**Total**
Surface-applied							
PL	226 a‡	270 b	6 a	502 a	4 a	33 a	37 a
PL + PC	83 b	376 a	17 a	476 a	6 a	42 a	49 a
PL + PH	95 b†	407 a†	17 †a	519 a†	4 a	39 a	43 a
Incorporated							
PL	97 a	254 b	7 a	358 a	36 a	37 a	73 a
PL + PC	39 b	323 ab	13 a	375 a	50 a	46 a	96 a
PL + PH	43 b	354 a	12 a	409 a	46 a	45 a	91 a

Legend for Table 2: PL refers to poultry litter; PC and PH refer to biochar from pine chips and peanut hulls, respectively.

‡ Within each column, different letters (a,b) mean significant difference between treatments at 0.05 level of significance;

† means one observation missing.

**Table 3.** Initial pH readings of poultry litter alone or in combination with soil, with or without acidified biochars.

**Sample**	**pH (without soil)**	**pH (with soil)**
PL	8.55 a†	6.94 a
PL + PC	7.26 c	6.47 b
PL + PH	7.39 b	6.73 ab

Legend for Table 3: PL refers to poultry litter; PC and PH refer to biochar from pine chips and peanut hulls, respectively.

† Within each column, different letters (a,b,c) mean significant difference between treatments at 0.05 level of significance.

**Table 4.** Inorganic N leached with 0.01 M CaCl_2_ from soil (64.5 g, s) and poultry litter (PL, 2.1 g) with or without acidified biochars (PC, PH, 2.1 g) surface-applied or incorporated.

**Treatments**	**Surface-applied**		**Incorporated**	
	**Leached NH_4_^+^**	**Leached NO_3_^−^**	**Leached NH_4_^+^**	**Leached NO_3_^−^**
	μg g ^−1^ soil			
PL	212 a‡	58 a	215 b	38 a
PL + PC	284 a	92 a	253 ab	70 a
PL + PH	338 a†	69 a†	278 a	77 a

Legend for Table 4: PC and PH refer to biochar from pine chips and peanut hulls, respectively.

‡ Within each column, different letters (a,b) mean significant difference between treatments at 0.05 level of significance.

† means one observation missing.
